# The MHC-I-dependent neoantigen presentation pathway predicts response rate to PD-1/PD-L1 blockade

**DOI:** 10.17305/bb.2024.11069

**Published:** 2024-11-26

**Authors:** Yuchen Zhang, Chen Yang, Yanchao Xu, Xiang Jiang, Jiajun Shi, Binghua Li, Decai Yu

**Affiliations:** 1 State Key Laboratory of Pharmaceutical Biotechnology, Division of Hepatobiliary and Transplantation Surgery, Department of General Surgery, Nanjing Drum Tower Hospital, The Affiliated Hospital of Nanjing University Medical School, Nanjing, China

**Keywords:** Checkpoint blockades, immunotherapy, response prediction, MHC-I pathway, tumor mutational burden, TMB

## Abstract

Immune checkpoint inhibitors produce durable antitumor effects in various cancers, but not all patients respond. High tumor mutational burden (TMB) is a known predictor of clinical benefit. In this study, we focused on the MHC-I-dependent neoantigen presentation pathway to enhance predictive capabilities beyond TMB. Using pan-cancer immunogenomic analyses of somatic mutation data from The Cancer Genome Atlas(TCGA) and The International Cancer Genome Consortium (ICGC), we analyzed 33 cancer types. Objective response rates (ORRs) to PD-1/PD-L1 inhibitors were evaluated in relation to immune characteristics, including TMB, neoantigen load, MHC-I gene expression, and CD8+ T cell fraction. Spearman’s rank correlation was used to assess these relationships. TMB showed the strongest correlation with ORR (*r* ═ 0.783, *P* ═ 2.17 × 10^−5^). However, integrating TMB, HLA-A expression, and CD8+ T cell fraction significantly improved predictive accuracy (*r* ═ 0.865, *P* ═ 1.80 × 10^−6^). Validation in external cohorts confirmed these findings, revealing notable differences in MHC-I pathway activity between responders and non-responders to immunotherapy. Our results demonstrate that the MHC-I antigen presentation pathway is strongly associated with response to PD-1/PD-L1 inhibitors. Importantly, combining antigen expression, processing, presentation, and recognition features provides superior predictive power compared to TMB alone. This integrated approach could improve treatment outcome predictions and advance personalized immunotherapy strategies.

## Introduction

PD-1/PD-L1 checkpoint blockades have revolutionized cancer treatment for multiple cancers [1, 2], but their efficacy varies significantly across different cancer types and individual patients [3]. Therefore, identifying biomarkers that predict response to PD-1/PD-L1 inhibitors is crucial. Several predictors have emerged, reflecting both the tumor immune microenvironment and tumor cell intrinsic features, including PD-L1 expression, tumor mutational burden (TMB), neoantigens, microsatellite instability, tumor-infiltrating immune cells [4], metabolic pathways [5], and peripheral blood biomarkers [6]. As the most widely adopted predictor, PD-L1 expression remains controversial due to its low prediction accuracy and dynamic variability [7]. TMB is an emerging biomarker that positively correlates with the objective response rate (ORR) to PD-1 inhibition [8–10]. Recently, TMB, as estimated from both tumor tissue DNA (tTMB) and circulating tumor DNA (bTMB), has shown potential as a biomarker for immunotherapy across multiple cancer types [9, 11–13]. Tumor cells with high TMB may generate more neoantigens, leading to an increase in cancer-fighting T cells in both the tumor microenvironment and the periphery. These neoantigens are processed and presented on the surface of tumor cells via the MHC-I antigen processing pathway, where they are recognized by CD8+ T cells, triggering antitumor immune responses [14]. The MHC-I pathway consists of four major steps: antigen expression, processing, presentation, and recognition [15–17]. However, TMB alone has moderate clinical value as a biomarker. While it is well established that TMB correlates with neoantigen generation, the extent of DNA damage may also influence immune responses through other mechanisms [18]. Furthermore, TMB reflects only the endogenous antigen generation level and does not account for antigen processing, presentation, or immune infiltration status. Therefore, multiple factors contributing to the activity of the MHC-I pathway may influence the response to immunotherapy [19]. In this study, we conducted pan-cancer immunogenomic analyses to identify novel biomarkers for PD-1/PD-L1 inhibitor-based immunotherapy.

## Materials and methods

### ORR in the literature

ORRs for PD-1 or PD-L1 inhibitors in major solid tumor types were reported by Yarchoan et al. [8]. Briefly, the authors conducted a search for articles and abstracts that reported ORRs for anti-PD-1 or anti-PD-L1 therapies and summarized the ORRs across different cancer types. The ORRs and patient numbers for the tumor types analyzed in the study are presented in Table S1.

### Data sources

Data from a total of 11,069 samples representing 33 cancer types with available RNASeqv2 data from The Cancer Genome Atlas (TCGA) portal were downloaded from PanCanAtlas (https://gdc.cancer.gov/about-data/publications/pancanatlas). It should be noted that while data from 33 cancer types were collected, only 21 had sufficient data for analyzing immunotherapy response rates. Gene expression values were represented as RNA-seq by expectancy maximization (RSEM) data and were normalized within each sample to the upper quartile of total reads. The mutation file, “mc3.v0.2.8.PUBLIC.maf.gz,” was also downloaded from PanCanAtlas. The International Cancer Genome Consortium (ICGC) dataset was downloaded from UCSC Xena (https://xenabrowser.net/datapages/).

The immune characteristics in each TCGA tumor sample were obtained from Thorsson et al. [20], with all quantitative immune-related indexes included in this analysis. To evaluate the correlations between ORR and antigen processing and presentation procedures, we analyzed the expression of MHC-I genes (HLA-A, HLA-B, and HLA-C) as well as a gene set reflecting endogenous antigen processing and presentation, named GO_ANTIGEN_PROCESSING_AND_PRESENTATION_OF_ENDOGENOUS_ANTIGEN (abbreviated as the GO_ANTIGEN gene set), from the Molecular Signatures Database (MSigDB). The median expression of all involved genes was used to represent the sample’s expression of the pathway.

Two publicly available datasets containing genetic data, transcriptomic information, immune checkpoint blockade (ICB) responsiveness, and clinical outcomes were used to validate the results: the Damrauer et al. cohort and the Liu et al. cohort. The Damrauer cohort comprised 147 patients with urothelial cancer who underwent DNA and RNA sequencing while receiving immune checkpoint inhibitors [21]. The Liu cohort included 121 patients with advanced melanoma treated with anti-PD-1 ICB, with both whole-exome sequencing (WES) and RNA sequencing performed [22]. Only patients with complete molecular profiling and clinical data were included in the analysis.

### TMB

TMB was defined as the number of somatic, coding, base substitution, and indel mutations per megabase of the genome examined. Mutation data from TCGA and ICGC were used to calculate TMB for each tumor sample, as previously described [23].

### Modeling the MHC-I pathway activity

The immune characteristics were quantile normalized before modeling to standardize their magnitudes. We selected immune features associated with antigen expression, processing, presentation, and recognition to model MHC-I pathway activity. Our analysis revealed that the combination of TMB, HLA-A expression, and the fraction of CD8+ T cells yielded the best predictive value. Our linear correlation formula is ORR ═ 8.82 (TMB) + 4.71 (HLA-A) + 1.62 (CD8+ T cells fraction) − 38.4.

### Validation of predictive value in external cohorts

Two external cohorts (the Damrauer et al. cohort and the Liu et al. cohort), consisting of patients receiving ICB therapy, were used to validate the predictive value of the MHC-I-dependent neoantigen presentation pathway in response to anti-PD-1/PD-L1 therapy. Receiver operating characteristic (ROC) curve analysis was performed to evaluate the predictive performance of TMB, HLA-A expression, CD8 levels, and MHC-I pathway activity in relation to anti-PD-1/PD-L1 response. The area under the curve (AUC) and ROC curve were calculated using the “roc” and “ggroc” functions from the “pROC” package.

### Statistical analysis

Correlations between immune characteristics and ORRs were assessed using Spearman’s rank correlation coefficient. TMB levels were compared between groups with the Wilcoxon rank-sum test. Progression-free survival (PFS) and overall survival (OS) were estimated using the Kaplan–Meier method, and comparisons between study groups were made with the log-rank test. The median value was used as the cut-off to categorize immune characteristics into dichotomous variables. Survival analysis results were visualized with a heatmap. All analyses were performed using R 3.5.1. A significance level of 0.05 was set for all tests, which were two-sided.

## Results

### The MHC-I pathway

**Figure 1.**
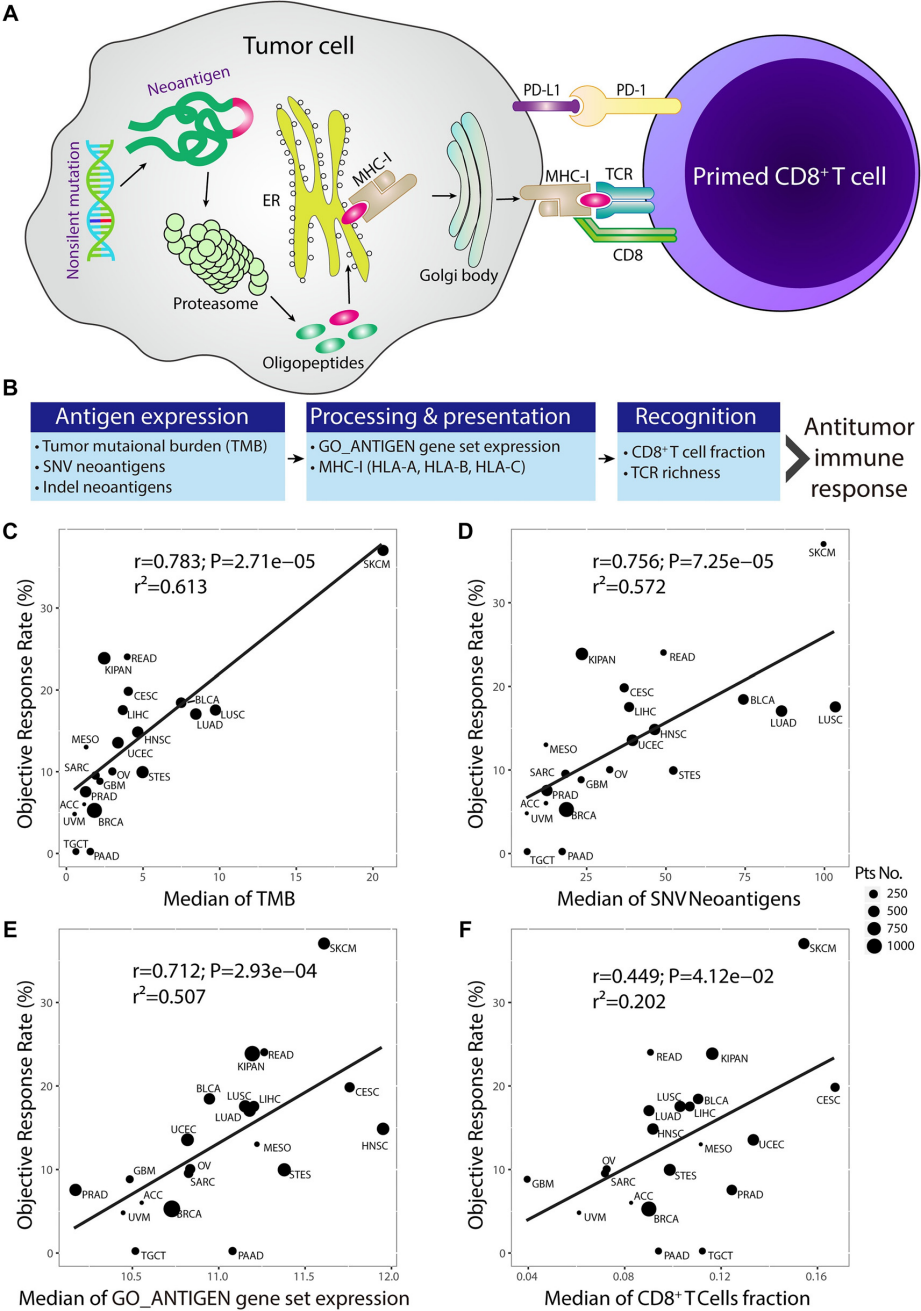
**The MHC-I presentation pathway of cancerneoantigens and correlations between itscomponents and ORRacross cancer typesin the TCGA dataset.** (A) Schematic model illustrating tumor neoantigens expression, processing, presentation, and recognition procedures in the MHC-I pathway. Nonsilent somatic mutation-derived neoantigens were expressed, processed by the proteasome into oligopeptides, and then translocated into the endoplasmic reticulum and presented on the cell surface by MHC-I from the Golgi body. Finally, Primed CD8^+^ T cells recognized the MHC-I/peptide complexes through their TCR; (B) Candidate ORR-related immune characteristics in the MHC-I pathway; (C) Correlation between TMB and ORR; (D) Correlation between SNV neoantigens and ORR; (E) Correlation between the median expression of the GO_ANTIGEN gene set and ORR; (F) Correlation between the median CD8^+^ T cells fraction and ORR across cancer types. TMB: Tumor mutational burden; TCGA: The Cancer Genome Atlas.

A high TMB typically results in more neoantigens, which enhances immunogenicity and likely increases CD8+ T-cell antitumor reactivity. Neoantigens are expressed, processed, and presented on tumor cells by MHC-I, a process that can sometimes trigger successful T-cell-dependent immune responses by activating neoantigen-specific CD8+ T cells. This mechanism is well understood as the MHC-I pathway (Figure 1A). It is also well established that TMB serves as an emerging biomarker for predicting clinical responses to PD-1 or PD-L1 inhibitors [8–10]. Based on this, we hypothesize that key components of the MHC-I pathway—encompassing antigen expression, processing, presentation, and recognition—such as TMB, neoantigens, the MHC-I gene family, the GO_ANTIGEN gene set, TCR richness, and the fraction of CD8+ T cells, may serve as candidate biomarkers for predicting the efficacy of PD-1/PD-L1 blockade therapies (Figure 1B).

**Figure 2.**
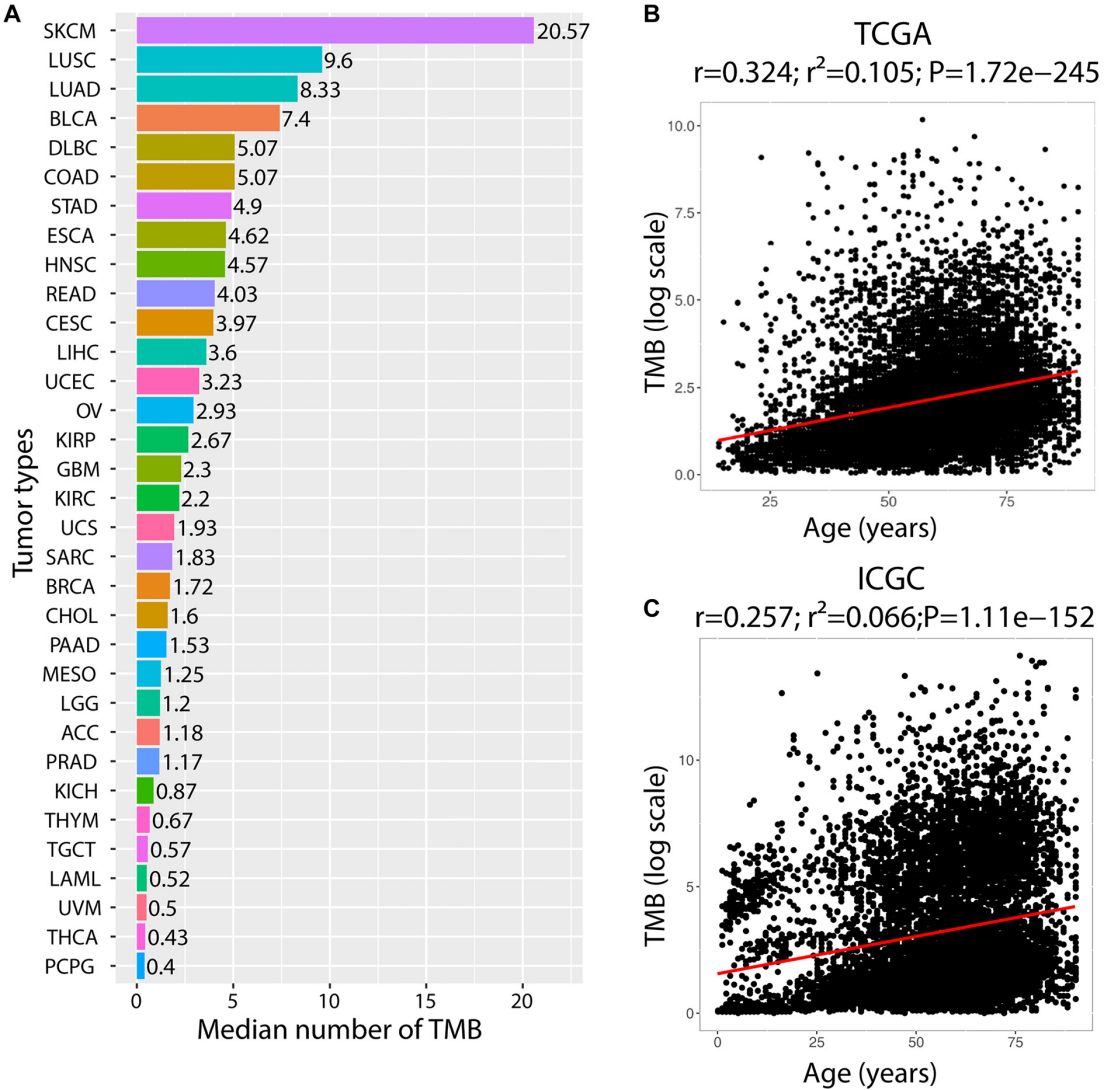
**The landscape of tumor mutation burden.** (A) Median numbers of tumor TMB within TCGA tumor types. Abbreviations of cancer types see the legend of Figure S2B. Correlations between TMB and age across cancer types in TCGA dataset (B) and ICGC dataset (C). TMB: Tumor mutational burden; TCGA: The Cancer Genome Atlas.

### Pan-cancer immunogenomic analyses

To identify immune indexes related to ORR, we plotted the ORR for anti-PD-1 or anti-PD-L1 therapy against immune characteristics across multiple cancer types (Table S2). Significant correlations were observed between ORR and various immune-related metrics, including TMB (*r* ═ 0.783, *P* ═ 2.71 × 10^−5^), silent mutation rate (*r* ═ 0.759, *P* ═ 6.65 × 10^−5^), nonsilent mutation rate (*r* ═ 0.749, *P* ═ 9.29 × 10^−5^), SNV neoantigens (*r* ═ 0.756, *P* ═ 7.25 × 10^−5^), Indel neoantigens (*r* ═ 0.644, *P* ═ 0.0029), HLA-A (*r* ═ 0.509, *P* ═ 1.83 × 10^−2^), HLA-B (*r* ═ 0.637, *P* ═ 1.93 × 10^−3^), HLA-C (*r* ═ 0.671, *P* ═ 8.79 × 10^−4^), GO_ANTIGEN gene set (*r* ═ 0.712, *P* ═ 2.93 × 10^−4^), and CD8+ T cell fraction (*r* ═ 0.449, *P* ═ 4.12 × 10^−2^) (Figure 1C–1F and Figure S1). These results demonstrate that key components of the MHC-I pathway, in addition to TMB, are correlated with the response to anti-PD-1 or anti-PD-L1 therapy. We also evaluated the prognostic value of ORR-related immune characteristics across multiple cancer types. The relationship between these immune characteristics and survival varied by cancer type. For instance, TMB was associated with worse PFS in several cancers, while CD8+ T cell fraction was linked to better OS and PFS in many cancer types (Figure S2A).

### The landscape of tumor mutation burden

We examined the landscape of TMB across 33 cancer types from the TCGA dataset. TMB varies dramatically among tumor types [23, 24], which reflects significant differences in the balance of DNA damage and DNA repair fidelity among tumors. We found that TMB was high in tumor types originating from organs directly exposed to (SKCM) or indirectly connected to the external environment with pipes (LUSC, LUAD, BLCA, COAD, STAD, ESCA, and UCEC), and low in tumor types derived from deep body tissues (PCPG, THYM, PRAD, PAAD, ACC, and LGG), indicating that TMB was a marker of genomic damage (Figure 2A). Given that somatic mutation accumulated with age [25], we further analyzed the correlations between TMB and age. TMB was positively correlated with age in both the TCGA dataset (*r* ═ 0.324, *P* ═ 1.7210^−245^, Figure 2B) and the ICGC dataset (*r* ═ 0.257, *P* ═ 1.1110^−152^, Figure 2C), and increased with age across most cancer types (Figure S2B). Collectively, these results illustrated that TMB was a marker of DNA damage, and that greater age contributed to higher TMB.

### The MHC-I pathway activity and response to immunotherapy

**Figure 3.**
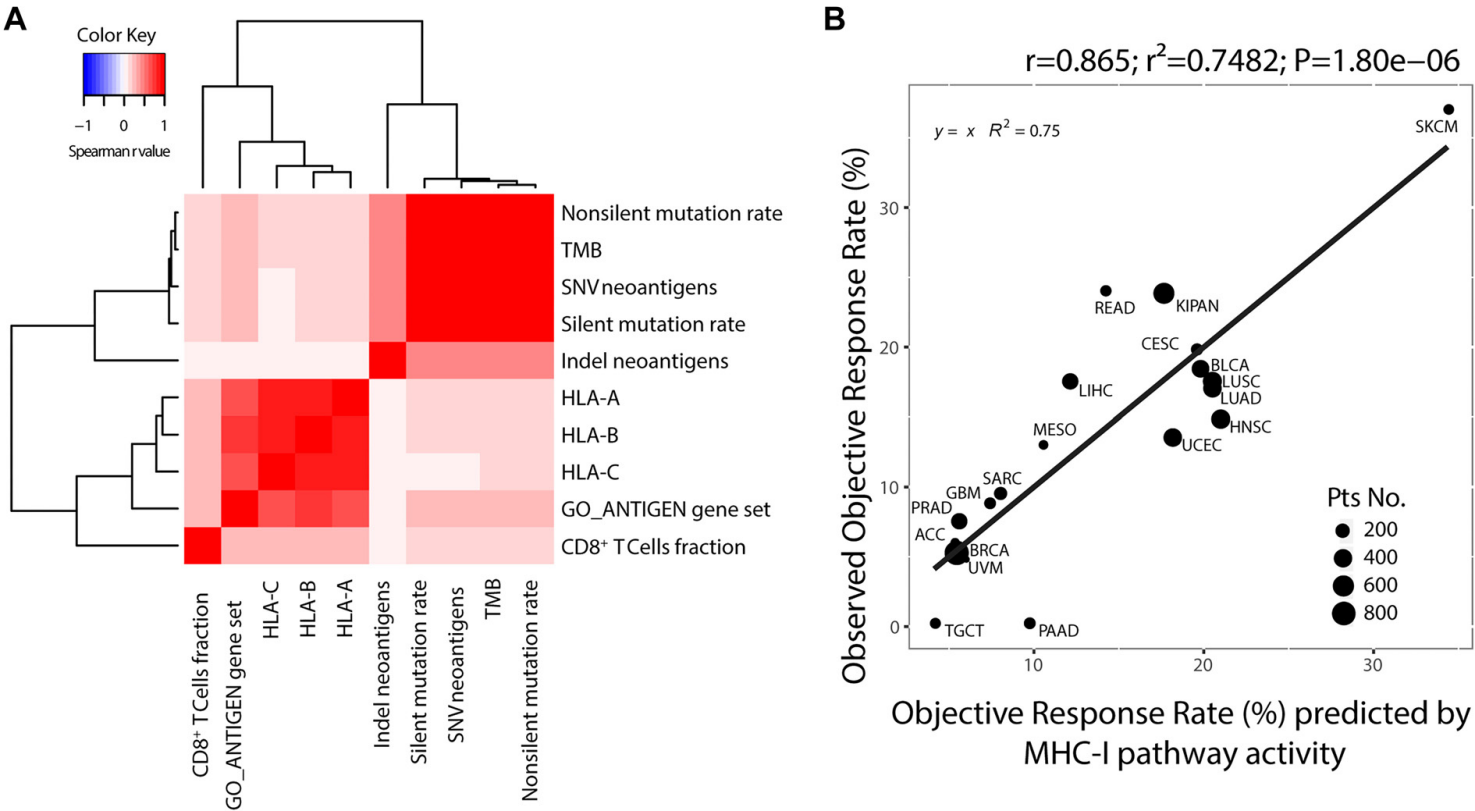
**TMB combined with antigen processing, presentation, and recognitionindexesimprove the accuracy of prediction.** (A) Cluster analysis of the ORR-related immune characteristics based on the Spearman correlation coefficient. Red indicates positive correlations and blue indicates negative correlations. (B) Correlation between the MHC-I pathway activity and ORR across cancer types. TMB: Tumor mutational burden.

To explore the relationships among ORR-related immune characteristics, we calculated the Spearman correlation coefficient matrix (Figure 3A and Figure S3). TMB and other indices related to antigen expression, including silent mutation rate, nonsilent mutation rate, and neoantigens, were strongly and positively correlated (*r* > 0.95), which validated our method for TMB calculation. Similarly, characteristics reflecting endogenous antigen processing and presentation showed a remarkable positive correlation. TMB and neoantigens serve as biomarkers indicative of antigen expression. However, TMB alone cannot fully reflect MHC-I pathway activity, as it overlooks antigen processing, presentation, and lymphocytic infiltration. Therefore, we selected immune features that capture antigen expression, processing, presentation, and recognition (referred to as MHC-I pathway activity) to predict ORR. We found that the combination of TMB, HLA-A expression, and CD8+ T cell fraction provided the best predictive value (*r* ═ 0.865, *P* ═ 1.80 × 10^−6^). This correlation coefficient of 0.865 suggests that 75% of the variance in ORR across cancer types may be explained by MHC-I pathway activity (Figure 3B).

### Validation of predictive value for immunotherapy response in external cohorts

Two external cohorts—the Damrauer cohort, comprising urothelial cancer patients, and the Liu cohort, consisting of advanced melanoma patients—were used to validate the predictive value of the MHC-I-dependent neoantigen presentation pathway in response to anti-PD-1/PD-L1 therapy. Responders to anti-PD-1/PD-L1 therapy exhibited higher TMB (*P* ═ 0.0063), HLA-A expression (*P* ═ 0.08), and CD8 T cell fraction (*P* ═ 0.0012) compared to non-responders. Although responders had higher levels of HLA-A expression than non-responders (*P* ═ 0.8), this difference did not achieve statistical significance, suggesting that, within the context of this study, HLA-A expression may not be a robust independent biomarker for predicting immunotherapy response.

**Figure 4.**
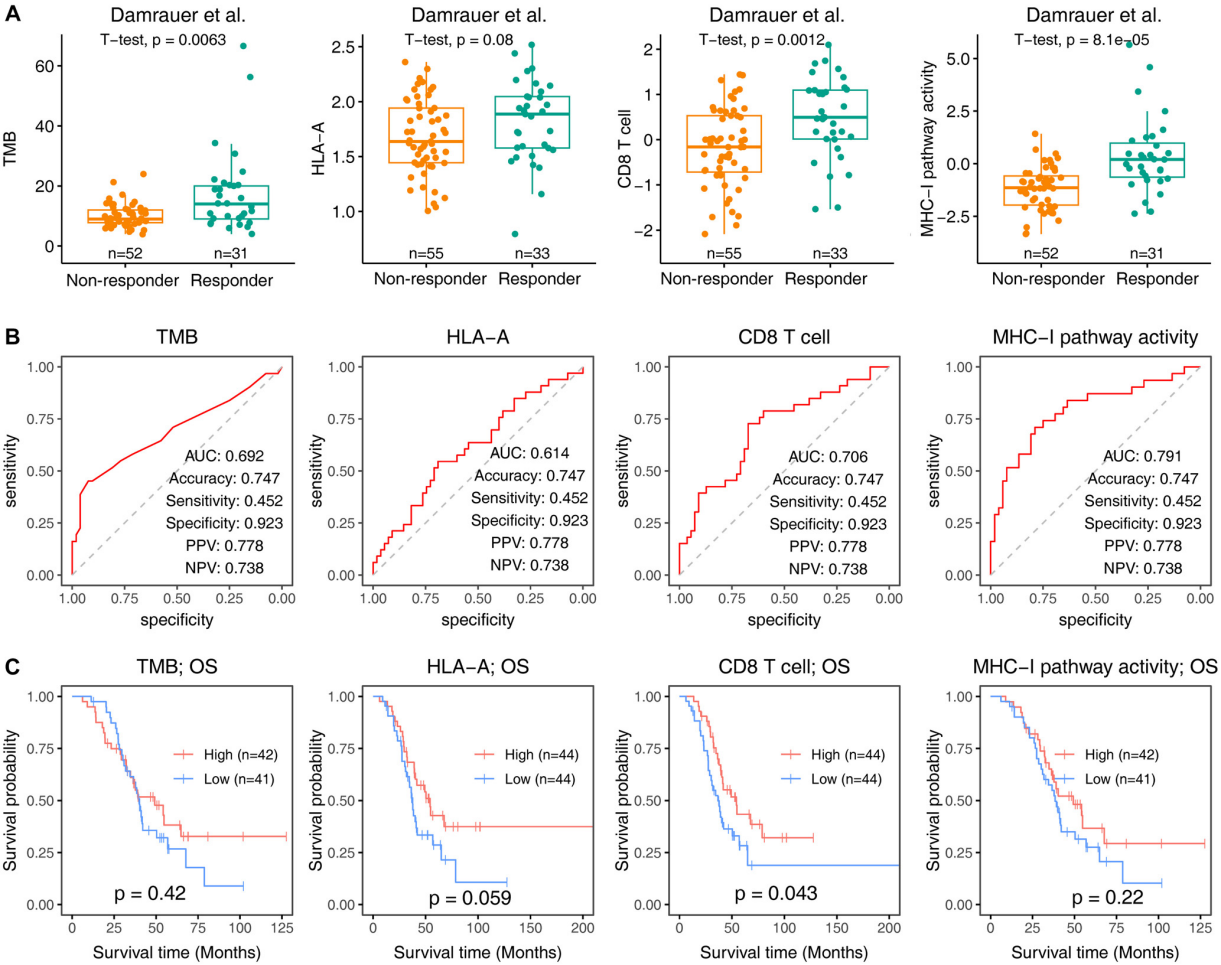
**Validation of the predictive value of the MHC-I-dependent neoantigen presentation pathway in response to anti-PD-1 therapy in urothelial cancer (Damrauer et al. cohort).** (A) Boxplot illustrating TMB, HLA-A expression, CD8+ T cell infiltration, and MHC-I pathway activity in anti-PD-1 therapy responders vs non-responders; (B) ROC curve demonstrating the predictive performance of TMB, HLA-A, CD8+ T cell infiltration, and MHC-I pathway activity for anti-PD-1 therapy response in the Damrauer et al. cohort; (C) Kaplan–Meier plots depicting OS in the Damrauer et al. cohort, stratified into high and low groups (split by median) based on TMB, HLA-A, CD8+ T cell infiltration, and MHC-I pathway activity. TMB: Tumor mutational burden; ROC: Receiver operating characteristic.

A more pronounced difference was observed in MHC-I pathway activity (*P* ═ 8.1e10^-5^), modeled using TMB, HLA-A, and CD8 T cells (Figure 4A). TMB, HLA-A, and CD8 predicted immunotherapy response with AUC values of 0.692, 0.614, and 0.706, respectively, while the MHC-I pathway showed a higher AUC of 0.791 (Figure 4B). Elevated HLA-A expression and CD8 T cell fraction correlated positively with OS, whereas TMB and MHC-I pathway activity were not significantly associated with OS in urothelial cancer.

In the Liu cohort, MHC-I pathway activity provided greater predictive value for immunotherapy response than TMB, HLA-A, or CD8 T cells alone (Figure S4A and S4B). In contrast to urothelial cancer, higher TMB or MHC-I pathway activity correlated positively with both OS and PFS in melanoma, while HLA-A expression and CD8 T cell fraction were not significant. This indicates heterogeneity across cancer types, consistent with the results shown in Figure S2A.

In conclusion, we validated that the MHC-I pathway is positively associated with response to PD-1/PD-L1 blockades in two external datasets.

## Discussion

In this study, we performed pan-cancer immunogenomic analyses to identify biomarkers associated with clinical responses to checkpoint blockade immunotherapy. Our findings suggest that key components of the MHC-I pathway—including antigen expression, processing, presentation, and recognition—are positively correlated with the ORR to PD-1/PD-L1 immunotherapy. Several elegant studies have demonstrated that TMB may influence the efficacy of immune checkpoint inhibitors [9, 13]. WES and targeted next-generation sequencing have enabled comprehensive characterization of antigen-related indices, such as TMB and neoantigens in tumor samples [26, 27]. However, TMB alone does not account for the tumor’s antigen processing and presentation capacity or the infiltration of immune cells [28]. We identified several easily accessible biomarkers, including MHC-I gene expression and CD8+ T cell fraction, which may predict clinical benefit from PD-1/PD-L1 blockade. Recent studies have underscored the importance of MHC-I expression in overcoming resistance to cancer immunotherapy. For example, high expression of MHC Class I has been shown to bypass immunotherapy resistance linked to defects in the IFNγ signaling pathway [29]. This aligns with our finding that MHC-I pathway activity could explain 75% of the variability in ORR across cancer types. Moreover, targeting cathepsin B with cycloastragenol has been shown to enhance CD8+ T cell-mediated antitumor immunity by inhibiting MHC-I degradation [30], further supporting the pivotal role of MHC-I in modulating immune responses to cancer. In the case of urothelial carcinoma, MHC-I expression has been identified as a predictor of response to checkpoint inhibitors in metastatic disease, though it lacks prognostic value in localized disease [31]. This suggests that the role of MHC-I in immunotherapy response may vary by cancer type and disease stage. Additionally, a recent study found that both MHC-I genotype and TMB could predict immunotherapy responses [19]. This complements our observation that integrating antigen expression, processing, presentation, and recognition features offers superior predictive performance compared to TMB alone. Interestingly, systemic interferon-γ has been shown to increase MHC Class I expression and T cell infiltration in cold tumors, as demonstrated in a phase 0 clinical trial [32]. This provides further evidence that modulating MHC-I expression could enhance the efficacy of immunotherapy.

The correlation coefficient between TMB and ORR is 0.74, suggesting that TMB accounts for 55% of the variability in ORR across different cancer types [8]. However, our study found a slightly higher correlation coefficient of *r* ═ 0.783, indicating a stronger association between TMB and ORR in our sample. Despite this, when used as a standalone predictive marker, TMB does not significantly correlate with ORR in a substantial portion of cancer patients [33]. Additionally, studies have shown that there is no clear positive correlation between neoantigen burden and CD8+ T cell infiltration in tumors [34]. In our study, we found that the correlation coefficient increased to 0.865 when combining TMB, HLA-A expression, and CD8+ T cell fraction. This suggests that MHC-I pathway activity can explain 75% of the differences in ORR across cancer types. In external cohorts, we validated that, compared to non-responders, anti-PD-1/PD-L1 responders exhibited higher TMB (*P* ═ 0.0063), an increase in HLA-A expression levels that did not reach statistical significance (*P* ═ 0.08), and an improved CD8 T cell ratio (*P* ═ 0.0012). Furthermore, we found that the differences in MHC-I pathway activity, as modeled by TMB, HLA-A expression, and CD8 T cell ratio, were more pronounced than those for TMB, HLA-A, or CD8 T cell ratio alone (*P* ═ 8.1e-5). We also examined the landscape of TMB across 33 cancer types. TMB was generally higher in tumors derived from superficial organs compared to deep-seated tissues, and high TMB was consistently associated with poorer survival [33]. This suggests that while TMB is associated with clinical benefits and sensitivity to anti-PD-1/PD-L1 therapy, it may also indicate high DNA damage, more aggressive tumor behavior, and poorer prognosis for patients. Additionally, we found a significant positive correlation between TMB and age (*r* ≈ 0.3), consistent with existing research. This implies that age may be a potential confounding factor when defining the optimal cut-off value for TMB [24]. There are several limitations to our study. First, it is retrospective, and only 21 cancer types were analyzed for immunotherapy response. Additionally, the tumor specimens used for sequencing were not the same ones from which ORR was calculated. Second, while we proposed that age might influence TMB, we were unable to precisely determine the impact of age on TMB or define cut-off values for different age groups. Third, while we observed a correlation between MHC-I pathway activity and ORR, the lack of uniform quantitative criteria and challenges in defining cut-off values limit its clinical applicability. Finally, our results should be validated in larger, prospective cohorts. In conclusion, our study suggests that MHC-I pathway activity is positively associated with the response to PD-1/PD-L1 blockades. Integrating antigen expression, processing, presentation, and recognition characteristics yields better predictive efficiency than TMB alone. TMB is also positively correlated with age, indicating that age should be considered when defining TMB cut-off values. These findings warrant further investigation in larger solid tumor cohorts receiving immune checkpoint therapies.

## Supplemental data

Supplemental data are available at the following link: https://www.bjbms.org/ojs/index.php/bjbms/article/view/11069/3629.

## Data Availability

The data that support this study are available from the corresponding author upon reasonable request.
